# Reliability and reproducibility analysis of the Cobb angle and assessing sagittal plane by computer-assisted and manual measurement tools

**DOI:** 10.1186/1471-2474-15-33

**Published:** 2014-02-06

**Authors:** Weifei Wu, Jie Liang, Yuanli Du, Xiaoyi Tan, Xuanping Xiang, Wanhong Wang, Neng Ru, Jinbo Le

**Affiliations:** 1 Department of Orthopedics, the People’s Hospital of Three Gorges University, the First People’s Hospital of Yichang, Yichang, China

**Keywords:** SurgimapSpine software, Manual tool, Sagittal parameters measurement, Differences

## Abstract

**Background:**

Although many studies on reliability and reproducibility of measurement have been performed on coronal Cobb angle, few results about reliability and reproducibility are reported on sagittal alignment measurement including the pelvis. We usually use SurgimapSpine software to measure the Cobb angle in our studies; however, there are no reports till date on its reliability and reproducible measurements.

**Methods:**

Sixty-eight standard standing posteroanterior whole-spine radiographs were reviewed. Three examiners carried out the measurements independently under the settings of manual measurement on X-ray radiographies and SurgimapSpine software on the computer. Parameters measured included pelvic incidence, sacral slope, pelvic tilt, Lumbar lordosis (LL), thoracic kyphosis, and coronal Cobb angle. SPSS 16.0 software was used for statistical analyses. The means, standard deviations, intraclass and interclass correlation coefficient (ICC), and 95% confidence intervals (CI) were calculated.

**Results:**

There was no notable difference between the two tools (*P* = 0.21) for the coronal Cobb angle. In the sagittal plane parameters, the ICC of intraobserver reliability for the manual measures varied from 0.65 (T2–T5 angle) to 0.95 (LL angle). Further, for SurgimapSpine tool, the ICC ranged from 0.75 to 0.98. No significant difference in intraobserver reliability was found between the two measurements (*P* > 0.05). As for the interobserver reliability, measurements with SurgimapSpine tool had better ICC (0.71 to 0.98 vs 0.59 to 0.96) and Pearson’s coefficient (0.76 to 0.99 vs 0.60 to 0.97). The reliability of SurgimapSpine measures was significantly higher in all parameters except for the coronal Cobb angle where the difference was not significant (*P* > 0.05).

**Conclusion:**

Although the differences between the two methods are very small, the results of this study indicate that the SurgimapSpine measurement is an equivalent measuring tool to the traditional manual in coronal Cobb angle, but is advantageous in spino-pelvic measurement in T2-T5, PT, PI, SS, and LL.

## Background

The Cobb method based on image remains the most important technique for assessment of spinal deformity in both coronal and sagittal planes worldwide [1-5]. The Cobb angle has been used to choose the type of treatment, to evaluate progression of the curve, and to appraise the effectiveness of treatment [6,7]. Treatments are selected according to the degree of curvature or the progression of the curve beyond definite amounts. Because the effects of the treatment are significant, it is important that the reliability of the measurements be well recorded to avoid major underestimation or overestimation of the changes that can be produced by observer error.

The intraobserver and interobserver reliability of the Cobb angle measured by different techniques such as manual and smart phone for coronal plane deformity has been well studied [3,8]. For quantitative measurements of curvature, a study carried out with manual and digital measurement tools in 48 patients with scoliosis concluded that digital radiography did not improve the measurement accuracy [8]. However, there are a few studies about the measurement of pelvic morphology [9,10] but no study based on computer-ancillary techniques as well as SurgimapSpine software method was found. SurgimapSpine software technique to measure Cobb angle is the most popular method used in our studies. Its reliable and reproducible measurements as well as their accurate communications are critical for clinical studies. Therefore, the aims of current study are to assess the interobserver and intraobserver reliability of Cobb angle measured by manual and computer-ancillary techniques using coronal and sagittal planes radiographs, and to compare the differences between the two methods.

## Methods

### Subjects

From February 2011 to January 2013, radiographs satisfying the following conditions were included in this study: Cobb angle not above 90° because large Cobb angle is often associated with vertebral superimposed image, no obvious thoracic kyphosis, T2, T5, and pelvis being seen clearly. All X-rays were printed for manual measurements, and the cranial and caudal end vertebrae were marked by the senior spine surgeon on the same radiographs to reduce the component of variability. This study was approved by the clinical research ethics committee of the People’s Hospital of Three Gorges University. Informed consent for data analysis was obtained from all subjects and/or families.

Three examiners, all orthopedic surgeons familiar with the measurement method of the Cobb angle, carried out the measurements independently in each setting (manual measurement on radiographs and SurgimapSpine software ancillary measurement on the computer). Each observer measured each radiograph twice, with a week’s interval between the first and second readings. All observers were blinded to their prior measurements and to the other observers. There is a learning curve for measurement of the Cobb angle on the computer. However, because SurgimapSpine method is being routinely used in the authors’ hospital since 2011, all the observers participating in the current study had already used this technique for at least a year.

**Figure 1:**
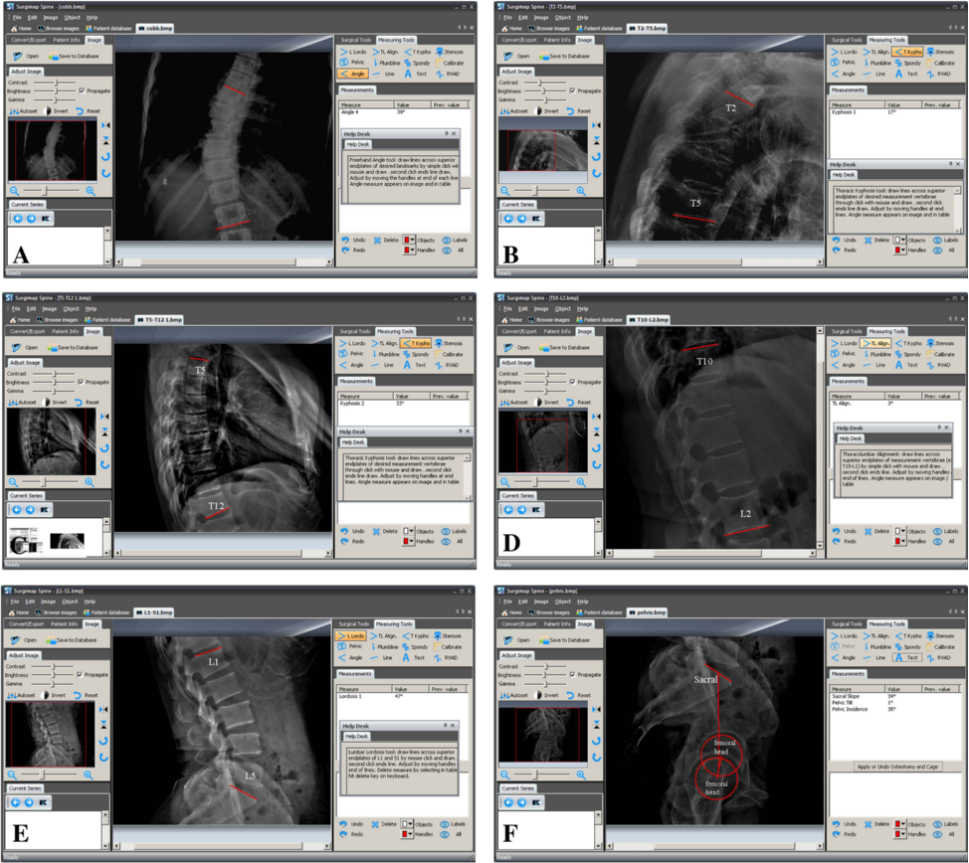
**Cobb angle and sagittal parameters measured with SurgimapSpine tool, part of the spine is enlarged and the contrast changed. A** is the measurement method of coronal Cobb angle; **B** and **C** is the measurement methods of T2–T5 and T5–T12, respectively; **D** and **E** is the thoracolumbar junction (TLJ: T10–L2) and lumbar lordosis (LL: L1–S1), respectively; **F** are the measurement methods of pelvis including pelvic incidence (PI), sacral slope (SS) and pelvic tilt (PT).

**Figure 2:**
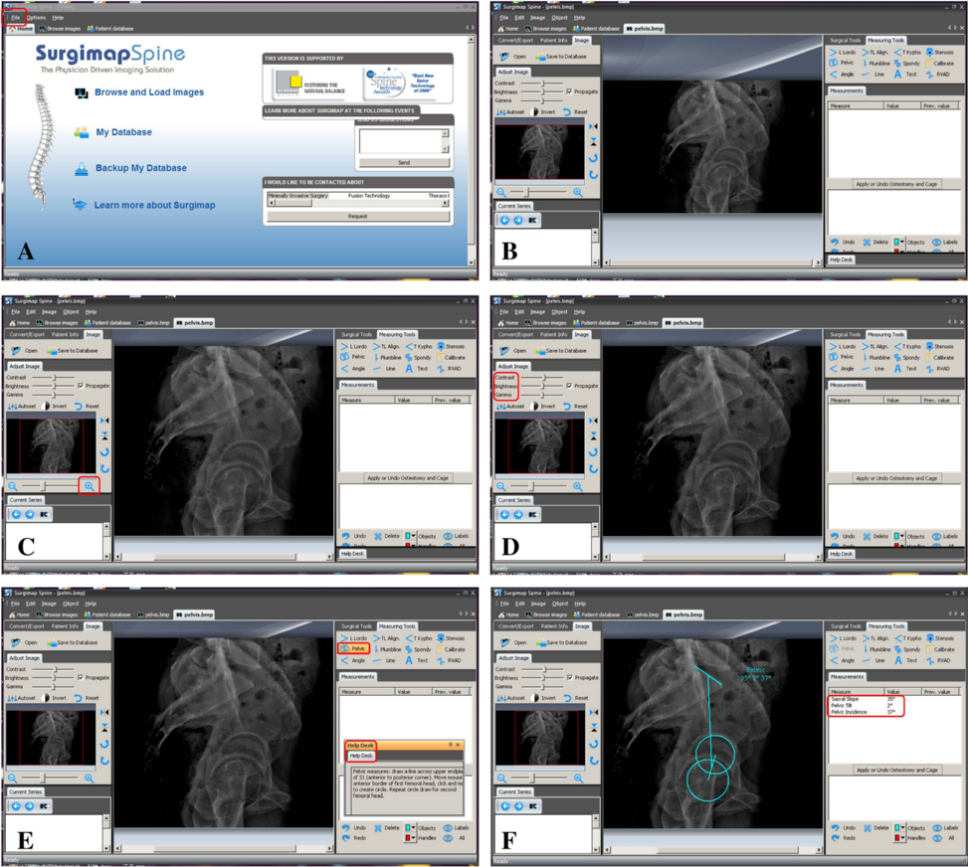
**Measuring methods of the software. A** is the main window when opening the SurgimapSpine software, the objects to be measured can be searched. **B** shows that the dimension of the image can be adjusted. **C** shows that the contrast of the image can be adjusted. **D** and **E** are the introductions and the specific measuring methods of pelvis measurement, respectively and **F** shows the measuring results displayed on the right side.

For the manual set, the main angle was measured with pencil, the same ruler, and protractor with standard methods as shown Figure 1. All radiographs were blinded and numbered consecutively. No copies were used to avoid the loss of quality as a result of duplication. Therefore, when one observer completed the measurement, the radiographs were wiped clean and passed to the next observer. For the specific software technique, all images were stored in the designated computer. The radiographs were all blinded, numbered, and viewed on the same SurgimapSpine software. Six parameters including coronal and sagittal planes were measured with manual and SurgimapSpine methods, respectively. Those measurements included pelvic incidence (PI), sacral slope (SS), pelvic tilt (PT), Lumbar lordosis (LL), thoracic kyphosis (T2–T5, T5–T12), and coronal Cobb angle [11]. The methods of parameters’ measurement are seen in Figure 1. As for operating methods of the software, the introductions and specific measuring methods exist with Cobb angle measurement in the same window, and the measuring results are displayed below the introductions on the right side (Figure 2). With regard to more than one curve in a patient, only the largest Cobb angle measured by observers was used in the final analysis.

Statistical analyses were performed using SPSS 16.0 software (SPSS Inc., Chicago, IL, USA). The means, standard deviations, intraclass and interclass correlation coefficient (ICC) (two-way mixed model, absolute agreement), 95% confidence intervals (CI) between the three observers, and between the two measurements of each observer were calculated. The ICC values can be considered as poor (less than 0.40), fair (0.40–0.59), good (0.60–0.74), and excellent (0.75–1.00) [12]. The level of significance was set at 0.05.

## Results

A total of 68 radiographs were chosen from among 100 radiographs of patients with scoliosis. For coronal Cobb angle, the range of variation was from 3.5° to 7.2° for manual measures and 3.2° to 6.1° for SurgimapSpine measures, the intraobserver reliability of the manual measures was from 0.93 to 0.95, and SurgimapSpine measures was from 0.94 to 0.96 (Table 1). When assessed by Pearson’s coefficient, there was no significant difference when compared with the ICC (Table 2) between the two methods. As for the intraobserver reliability, no notable difference was found between ICC and Pearson’s coefficient (Table 1).

**Table 1:** Intraobserver reproducibility of the manual and SurgimapSpine Cobb techniques for each observer based on intraclass correlation coefficients (95% confidence interval) and Pearson’s coefficient

		**Manual measurement**		**SurgimapSpine measurement**	
		**ICC(95% CI)**	**Pearson’s coefficient**	**ICC(95% CI)**	**Pearson’s coefficient**
Coronal Cobb angle	1	0.94(0.91–0.97)	0.95	0.94(0.89–0.96)	0.95
	2	0.93(0.84–0.96)	0.95	0.94(0.91–0.96)	0.94
	3	0.95(0.83–0.98)	0.97	0.96(0.90–0.98)	0.97
T2–T5	1	0.65(0.48–0.76)	0.66	0.75(0.63–0.84)	0.76
	2	0.68(0.53–0.79)	0.69	0.81(0.71–0.88)	0.81
	3	0.71(0.57–0.81)	0.74	0.76(0.64–0.85)	0.77
T5–T12	1	0.90(0.82–0.95)	0.92	0.91(0.84–0.95)	0.92
	2	0.88(0.76–0.93)	0.90	0.93(0.88–0.95)	0.93
	3	0.87(0.74–0.93)	0.90	0.92(0.87–0.95)	0.92
T10–L2	1	0.93(0.88–0.95)	0.93	0.94(0.91–0.97)	0.95
	2	0.91(0.86–0.94)	0.91	0.94(0.91–0.96)	0.94
	3	0.91(0.85–0.94)	0.91	0.96(0.93–0.97)	0.96
LL	1	0.92(0.88–0.95)	0.93	0.98(0.97–0.99)	0.98
	2	0.90(0.83–0.95)	0.93	0.93(0.89–0.96)	0.93
	3	0.95(0.89–0.97)	0.96	0.95(0.91–0.97)	0.95
PI	1	0.85(0.77–0.90)	0.85	0.87(0.80–0.92)	0.88
	2	0.82(0.48–0.92)	0.88	0.91(0.85–0.94)	0.91
	3	0.86(0.50–0.95)	0.92	0.88(0.58–0.95)	0.93
SS	1	0.89(0.83–0.93)	0.89	0.93(0.89–0.96)	0.93
	2	0.91(0.86–0.95)	0.92	0.92(0.88–0.95)	0.92
	3	0.92(0.84–0.95)	0.90	0.91(0.85–0.94)	0.92
PT	1	0.70(0.55–0.80)	0.70	0.74(0.62–0.83)	0.74
	2	0.70(0.49–0.82)	0.74	0.79(0.68–0.87)	0.79
	3	0.74(0.57–0.84)	0.78	0.77(0.66–0.85)	0.79

ICC: intraclass correlation coefficient; CI: confidence interval; PI: pelvic incidence; PT: pelvic tilt; SS: sacral slope; LL: lumbar lordosis; T: thoracic.

**Table 2:** Pearson correlation coefficients and significant differences between manual and SurgimapSpine Cobb measurement

**Parameters**	**Pearson’s coefficient**	***P value***
Coronal Cobb angle	0.98	0.21
T2–T5	0.59	0.005
T5–T12	0.90	0.002
T10–L2	0.98	0.000
LL	0.97	0.000
PI	0.84	0.002
SS	0.94	0.012
PT	0.68	0.012

PI: pelvic incidence; PT: pelvic tilt; SS: sacral slope; LL: lumbar lordosis; T: thoracic.

As for the sagittal plane parameters (T2–T5, T5–T12, T10–L2, LL, PI, PT, SS), the range of variation for manual methods was from 4.6° to 9.3°, 3.3 to 7.4, 2.1 to 6.5, 3.8 to 6.6, 2.8 to 7.9, 3.2 to 6.9, and 2.9 to 6.1, respectively. The range of variation for the SurgimapSpine measures was from 3.8° to 7.0°, 4.0 to 6.0, 2.0 to 4.4, 3.5 to 5.5, 2.5 to 6.6, 3.5 to 5.8, and 2.3 to 5.0, respectively. The intraobserver reliability for the manual measures varied from a low ICC of 0.65 (0.48–0.76) for determining the T2–T5 angle to a high of 0.95 (0.89–0.97) for determining the LL angle. In addition, for the SurgimapSpine tool, the ICC of the intra-observer reliability ranged from 0.75 (0.63-0.84) for the T2-T5 angle to 0.98 (0.97-0.99) for the LL angle. Whichever technique was used, the highest and lowest ICC was for the LL angle and T2–T5 angle, respectively. When using Pearson’s coefficient, the intraobserver reliability showed the same variability for the manual measures of sagittal plane parameters with as low as 0.66 for determining the T2–T5 angle to as high as 0.96 for determining the LL. With SurgimapSpine tool as well as the manual tool, the intraobserver reliability of LL was the best and that of PT the worst (Table 1). Overall, the intraobserver reliability of SurgimapSpine tool was obviously better than the manual tool in the measurement of sagittal plane parameters. However, no significant difference in intraobserver reliability was found between the two measurements.

The interobserver reliability for the manual measures varied from a low ICC of 0.59 for the PT angle and T2–T5 to a high of 0.96 for the T10–L2 angle. The interobserver reliability for the manual measures also varied from a low Pearson’s coefficient of 0.60 for the T2–T5 angle to a high of 0.97 for the T10–L2 angle (Table 3). When these values were compared to measures of reliability of SurgimapSpine measurements, it showed measurements with SurgimapSpine tool had better ICC ranging between 0.71 and 0.98 and Pearson’s coefficient ranging from 0.76 to 0.99 (Table 3).

**Table 3:** Interobserver reproducibility of the manual and SurgimapSpine Cobb techniques based on intraclass correlation coefficients (95% confidence interval) and Pearson’s coefficient

**Measuring methods**	**Coronal angle**	**Cobb**	**T2-5**		**T5-12**		**T10-L2**		**LL**		**PI**		**SS**		**PT**	
	**ICC(95% CI)**	**PC**	**ICC(95% CI)**	**PC**	**ICC(95% CI)**	**PC**	**ICC(95% CI)**	**PC**	**ICC(95% CI)**	**PC**	**ICC(95% CI)**	**PC**	**ICC(95% CI)**	**PC**	**ICC(95% CI)**	**PC**
Manual Cobb	0.96 (0.93-0.97)	0.96	0.59 (0.46-0.71)	0.60	0.87 (0.79-0.92)	0.90	0.96 (0.94-0.98)	0.97	0.95 (0.92-0.97)	0.96	0.77 (0.68-0.85)	0.79	0.94 (0.92-0.96)	0.94	0.59 (0.46-0.71)	0.61
																
Surgimap Spine Cobb	0.98 (0.96-0.98)	0.98	0.77 (0.68 0.85)	0.79	0.92 (0.86-0.95)	0.93	0.98 (0.96-0.99)	0.99	0.98 (0.96-0.99)	0.99	0.88 (0.83-0.92)	0.89	0.95 (0.93-0.97)	0.96	0.71 (0.64-0.82)	0.76

Pearson’s coefficients comparing SurgimapSpine measures to manual measures showed a low coefficient of 0.59 for the T2–T5 angle and a high coefficient of 0.98 for coronal angle and the T10–L2 angle (Table 3). The test of significant differences between reliability coefficients showed that the reliability of SurgimapSpine measures was significantly higher in all parameters except for coronal Cobb angle where the difference between the manual measures and the SurgimapSpine measures was not significantly different (Table 2).

## Discussion and conclusions

Treatment methods in patients with scoliosis depend on the Cobb angle in coronal plane and morphology of the sagittal planes [11,13-16]. Therefore, a veracious measurement is pivotal for the options of treatment. The Cobb angle measurement in the coronal plane has been studied fully, and the accuracy and reliability were good. Owing to variable measurement criteria, manual measurement errors, and difficulty in visualizing measurement landmarks in the measurement of the spina-pelvic alignment, the accuracy and reliability is often difficult and poor as previous studies demonstrated [9,10]. Therefore, developing a reliable method of radiographic measurement of the sagittal–pelvic alignment other than the traditional manual method is indispensable.

Computer-based SurgimapSpine measurement technique can open plain radiographs photographed or scanned, which form the digitized image. It adjusts image contrast and brightness enabling a better identification of key anatomical parameters not normally available for measurement on traditional radiographs. SurgimapSpine measurement technique has some advantages such as the following: rapid comparison between radiographs taken at different times of a patient, cheap storage, and images formatted by photos not films. In the present study, we found no significant difference in the intraobserver and interobservers’ reliability between the manual and the SurgimapSpine methods in the coronal Cobb’s angle measurement. However, the intra/interobserver reliability of the sagittal alignment found in SurgimapSpine tool was significantly better than those in manual method, especially in T2–T5, PI, and PT. Our data showed that the reliability of both the intraobserver and interobservers match well with the SurgimapSpine method and is more reliable in the Cobb angle measurement in the sagittal plane. When SurgimapSpine software is used for Cobb angle measurement, important parts of the spine can be enlarged and seen more clearly by changing the contrast, and the borders of the vertebrae can be enhanced by computerized options; after drawing lines through the endplates of end vertebrae, the software measures the angle automatically, which may reduce sources of error. Therefore, Cobb angle measurement by SurgimapSpine software both in coronal and sagittal alignment may be more accurate when compared with those measured with the manual method.

The coronal Cobb angle is usually used for the assessment and treatment of scoliosis. The excellent overall reliability of Cobb angle measurement has been well studied [1-3,8,10,17]. For the undefined end vertebra setting, Gstoettner [8] found a mean ICC of 0.97 for the intraobserver and interobserver reliability measurement by the manual method, whereas for the computer-assisted method, a mean ICC value for interobserver and intraobserver reliability was 0.93 and 0.96, respectively. Although measurement of Cobb’s angle using computer-assisted method was slightly better than that of the manual method, the computer-assisted method does not improve the measurement accuracy. Our study found similar excellent levels of intraobserver and interobserver reliability for the Cobb measurement by both manual and SurgimapSpine methods. These data suggested that the use of SurgimapSpine measurements does not improve measurement accuracy of the Cobb angle. In the end vertebrae defined setting, ICC of coronal Cobb angle in the current study was comparable to previous results [8,18] in which the end vertebrae were undefined, suggesting that the end vertebral selection was not an important factor in reliability of the Cobb measurement. Although different end vertebrae may result in Cobb angle variability, they do not influence accuracy of measurement. Therefore, in clinical practice it is not necessary to ensure the same end vertebrae.

In a study on sagittal–pelvic measurement for 29 normal young adults, John et al. [9] found the intraobserver ICC obtained by manual measures for PI was 0.69, PT 0.60, SS 0.77, and LL 0.90, and the ICC for interobserver was 0.41, 0.42, 0.64, and 0.57, respectively. Pearson correlation coefficient between computer-aided measures and manual measures for PI was 0.59, PT 0.63, SS 0.72, and LL 0.68. The authors concluded the reliability of computer-aided measures was notably higher in all parameters except for LL where the difference between the manual measures and the computer-aided measures was not obviously different. In the present study on AIS (adolescent idiopathic scoliosis) radiography using manual measurement, the reproducibility and reliability for T2–T5 and PT was only fair to good, whereas those angles of T5–T12, T10–L2, LL, PI, and SS were measured with excellent reproducibility both in intraobserver and interobserver. However, with regard to the intraobserver reproducibility and interobserver reliability of the SurgimapSpine tools, all parameters measured were excellent. Intra/interobserver reliability/reproducibility for T2–T5 thoracic kyphosis was markedly worse than for all other measures either in manual measures or in SurgimapSpine tool. Another study focusing on reliability of manual measures in AIS patients found intraobserver (0.22–0.83) and interobserver for T2–T5 (0.33–0.47) reliability was generally poor. However, other sagittal parameters were excellent [17]. The reliability and reproducibility of T2–T5 and PT Cobb angle measurement using both manual tool and SurgimapSpine tool in our study were disappointing. Other sagittal radiographic measures demonstrated good to excellent correlation. Causes for poor reliability of T2–T5 and PT Cobb angle may be related to the overlying density of the upper thoracic rib cage and scapula and femoral head. Our data also showed that sagittal measurement with SurgimapSpine tool obviously increases reproducibility and reliability, especially in Cobb angle of T2–T5 and PT. However, T2–T5 measurement is still not satisfactory.

Although the differences in the two methods are very small, the results of the present study indicate that the SurgimapSpine measurement is an equivalent measuring tool to the traditional manual in coronal Cobb angle, but is markedly advantageous in spino–pelvic measurement especially in T2–T5 and PT.

## Competing interests

The manuscript submitted does not contain information about medical drug(s). No funds were received in support of this work. No relevant financial activities outside the submitted work. The authors declare that they have no competing interests.

## Authors’ contribution

The authors report no conflict of interest concerning the materials or methods used in this study or findings specified in this paper. Author contributions to the study and manuscript preparation include the following. Conception and design: YD, WW. Parameters’ measurement: WW, JL, XT. Data analysis: JL, XX, WW, NR, JL. Drafting the article: YD, WW, JL, JL. Critically revising the article: YD, JL, WW, NR. Reviewed final version of the manuscript and approved it for submission: all authors. Study supervision: YD.

## Pre-publication history

The pre-publication history for this paper can be accessed here:

http://www.biomedcentral.com/1471-2474/15/33/prepub
